# Non-small cell lung cancer research: advances and persistent challenges

**DOI:** 10.3389/fonc.2026.1729747

**Published:** 2026-04-01

**Authors:** Lin Zhou, Jiakang Jiang

**Affiliations:** 1 Department of Internal Medicine, Daqing Hospital of Traditional Chinese Medicine, Daqing, China; 2 Department of Oncology, First Affiliated Hospital of Heilongjiang University of Chinese Medicine, Harbin, China

**Keywords:** acquired resistance, antibody-drug conjugates, biomarker validation, geriatric oncology, therapeutic accessibility

## Abstract

Non-small cell lung cancer (NSCLC) remains a leading cause of cancer-related mortality, prompting significant advancements in therapeutic and precision medicine. Recent innovations include antibody-drug conjugates (ADCs) such as TROP-2-targeting agents and HER3-DXd, which show promising efficacy in refractory disease. Next-generation tyrosine kinase inhibitors (TKIs), including lorlatinib, tepotinib, and glecirasib, have shown improved outcomes for patients with oncogene-driven NSCLC. Immunotherapy continues to evolve, with novel therapeutic targets and metabolic modulation strategies expanding its potential. Emerging diagnostic tools, such as liquid biopsy and artificial intelligence (AI)-based histopathology, are enhancing prognostic accuracy and enabling more personalized treatment approaches. Despite these advancements, significant challenges persist. Acquired resistance mechanisms and bypass pathways continue to undermine long-term therapeutic efficacy. Limitations in biomarker utility, including the imperfect predictive value of PD-L1and the lack of validation for ctDNA, STK11, and KEAP1, complicate treatment decision-making. While comprehensive genomic profiling (CGP) has expanded the detection of actionable targets, barriers such as accessibility, reimbursement issues, and workflow integration remain, with only 11-34% of eligible patients receiving matched therapies. Additionally, critical data gaps exist for elderly patients and rare subtypes such as hepatoid adenocarcinoma. Future efforts must prioritize overcoming resistance through combination strategies and ADCs, validating biomarkers using AI and ctDNA, streamlining CGP implementation, and addressing the unique needs of special populations. Bridging these biological and systemic challenges is essential for improving survival outcomes and ensuring equitable benefits for all NSCLC patients.

## 1 Introduction

Lung cancer remains a leading cause of cancer-related mortality worldwide, driving extensive research into novel therapeutic strategies and precision medicine approaches (1). The treatment landscape for non-small cell lung cancer (NSCLC) has rapidly expanded beyond traditional chemotherapy and initial targeted therapies. Current investigations are focused on overcoming resistance mechanisms, exploiting new molecular targets, refining immunotherapy applications, and addressing the unique needs of specific patient subgroups (2).

Significant progress has been made across multiple fronts. Antibody-drug conjugates (ADCs), such as those targeting trophoblastic cell surface antigen 2 (TROP-2), represent a promising frontier for refractory disease (3, 4). Simultaneously, next-generation TKIs for oncogenic drivers like ALK, ROS1, MET, and KRAS G12C offer improved efficacy (5–7). Diagnostic modalities are also evolving, with liquid biopsy for circulating tumor DNA (ctDNA) analysis and artificial intelligence (AI) applied to histopathology enhancing prognostic accuracy and personalization (8–10). Furthermore, comprehensive genomic profiling (CGP) is increasingly advocated to detect a broader range of actionable targets (11).

However, these advances are matched by persistent and multifaceted challenges. Acquired resistance to targeted agents and immunotherapies remains a fundamental biological hurdle (12). Biomarker validation, including for PD-L1, ctDNA dynamics, and mutations in STK11/KEAP1, lags behind therapeutic innovation (8, 10, 13). The clinical implementation of precision oncology faces systemic barriers, such as inconsistent access to testing, reimbursement issues, and workflow complexities, with only a minority of eligible patients receiving matched therapies (11, 14). Furthermore, robust data for special populations, including older adults and those with rare subtypes, remain scarce (15, 16).

**Figure 1:**
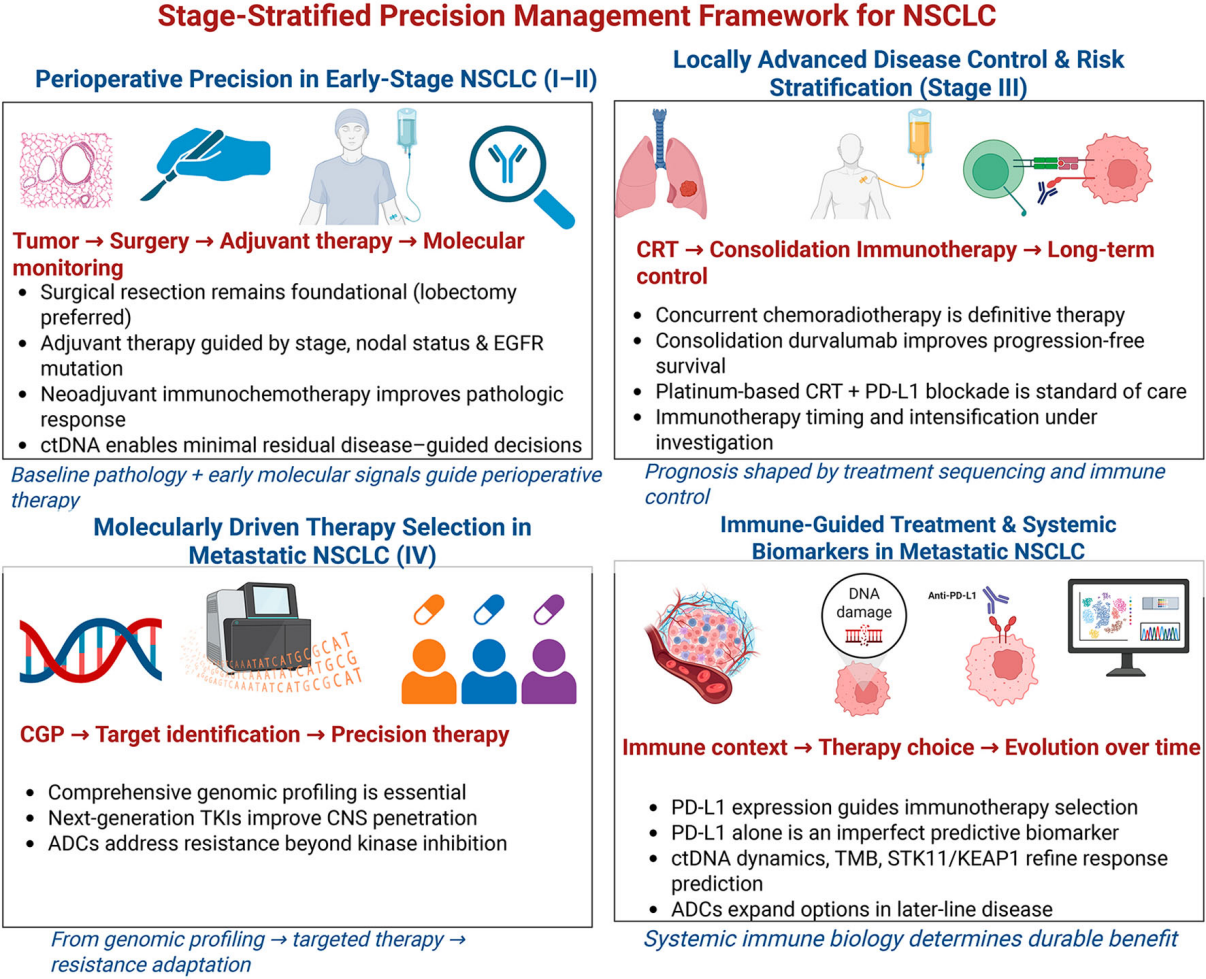
Stage-stratified precision management framework for non–small cell lung cancer. The schematic illustrates NCCN-aligned standard therapies and emerging biomarker-driven strategies across the NSCLC disease continuum. Early-stage disease emphasizes perioperative optimization through surgery, risk-adapted adjuvant or neoadjuvant therapy, and ctDNA-guided molecular monitoring. Locally advanced disease is managed with definitive concurrent chemoradiotherapy followed by consolidation immunotherapy to achieve durable disease control. In metastatic NSCLC, comprehensive genomic profiling enables molecularly driven targeted therapies, while immune-guided treatment selection incorporates PD-L1 expression and evolving systemic biomarkers. Together, this framework highlights the integration of pathology, genomics, and immune context to support precision, stage-appropriate clinical decision-making.

To synthesize these developments and challenges critically, this review is structured around three interconnected thematic axes: (1) Therapeutic innovations and resistance mechanisms, examining novel agents, their efficacy-toxicity profiles, and strategies to overcome resistance; (2) Diagnostic and biomarker advances, evaluating the integration and validation of tools like CGP, ctDNA, and AI; and (3) Implementation and equity challenges, addressing systemic barriers and evidence gaps in special populations. This reorganization aims to streamline the narrative, reduce redundancy, and provide a more analytical framework. Additionally, new algorithms and tables are incorporated to contextualize emerging therapies within current treatment paradigms and molecular testing workflows. By integrating this critical appraisal, the review aims to highlight actionable priorities for future research and clinical practice, guiding efforts to translate innovation into equitable improvements in NSCLC management. **Figure 1**; **Tables 1**, **2**.

**Table 1:** Outcomes from clinical trials evaluating therapies in specific patient populations.

Study objective	Methodology	Main findings	Conclusion	Reference
Assess afami-cel in HLA-A*02+ solid tumors	Phase 1 trial (38 patients)	ORR 24% (synovial sarcoma: 44%); Grade ≥3 hematologic toxicity; CRS (55%)	Efficacy in synovial sarcoma; Manageable safety	(17)
Compare osimertinib ± ramucirumab in EGFR+ NSCLC	Phase 2 RAMOSE trial (147 patients)	Median PFS 24.8 months vs. 15.6 months (HR 0.55); Grade ≥3 TRAEs: 53% vs. 41%	Ramucirumab-osimertinib improves PFS.	(18)
Evaluate glecirasib in KRAS G12C+ NSCLC	Phase 2b trial (119 patients)	ORR 47.9%; Grade ≥3 TRAEs 38.7%	Efficacy and manageable safety	(19)
Compare lorlatinib vs. crizotinib in ALK+ NSCLC	Phase III CROWN trial (296 patients)	Median PFS NR for lorlatinib vs. 9.1 months (HR 0.19); 5-year PFS 60% vs. 8%	Lorlatinib sets a new survival benchmark	(20)
Assess rezivertinib in first-line EGFR+ NSCLC	Phase 2a trial (43 patients)	ORR 83.7%; Median PFS 20.7 months; Grade ≥3 TRAEs 9.3%	Effective and safe in the first-line setting	(21)
Evaluate rezivertinib in EGFR T790M+ NSCLC	Phase 1/2 trial (172 patients)	ORR 59.3%; Median PFS 9.7 months	Safe and active in T790M+ NSCLC	(21)
Report tepotinib in METex14+ Japanese NSCLC	Phase 2 VISION subanalysis (38 patients)	ORR 60.5%; Median DoR 18.5 months; TRAEs: creatinine increase (65.8%)	Effective and safe in Japanese patients	(22)
Evaluate brigatinib in ROS1+ NSCLC	Phase 2 Barossa trial (47 patients)	ORR 71.4% (TKI-naïve) vs. 31.6% (post-crizotinib); Median PFS 12.0 vs. 7.3 months	Effective in TKI-naïve ROS1+ NSCLC	(23)
Compare furmonertinib vs. gefitinib in EGFR+ NSCLC	Phase 3 FURLONG trial (358 patients)	Median PFS 20.8 months vs. 11.1 months (HR 0.44); Grade ≥3 TRAEs: 11% vs. 18%	Furmonertinib superior to gefitinib	(21)
Evaluate KRAS G12C inhibitors in solid tumors	Systematic review of 17 trials	ORR: Sotorasib 7.1–47%, Adagrasib 19–53%; OS up to 24 months	KRAS G12C inhibitors improve responses	(24)
Evaluate iruplinalkib in ALK+ crizotinib-resistant NSCLC	Phase 2 INTELLECT trial (146 patients)	ORR 69.9%; Intracranial ORR 46%; Median PFS 14.5 months	Effective and safe in crizotinib-resistant NSCLC	(25)
Assess furmonertinib in EGFR T790M+ NSCLC	Phase 2b trial (220 patients)	ORR 74%; Median PFS 9.6 months; Grade ≥3 TRAEs 11%	Effective and safe post-TKI failure	(26)
Report patient-reported outcomes for tarlatamab in small-cell lung cancer	Phase 2 DeLLphi-301 trial (100 patients)	Stable QoL; Reduced dyspnea; Median OS 14.3 months; CRS (51%)	Maintains QoL in pretreated small-cell lung cancer	(27)

**Table 2:** First-line standard systemic therapy for metastatic NSCLC according to biomarkers.

Biomarker	Prevalence	Preferred first-line therapy	Alternative first-line options	Supporting evidence	Reference
EGFR mutation	10-15% (Caucasians) 40-55% (Asians)	Osimertinib (3rd gen TKI)	Erlotinib, Afatinib, Gefitinib, Dacomitinib, or Furmonertinib (in China)	Standard for common mutations (ex19del, L858R). CNS active. Resistance (e.g., C797S) remains a challenge.	(18, 20, 21)
ALK rearrangement	3-7%	Alectinib, Lorlatinib, or Brigatinib (Next-gen TKIs)	Crizotinib	Next-gen TKIs offer superior PFS and CNS control vs. crizotinib. Lorlatinib has highest reported 5-year PFS.	(3, 5, 20)
ROS1 rearrangement	1-2%	Entrectinib or Crizotinib	Lorlatinib, Repotrectinib	Entrectinib has superior CNS activity. Next-gen TKIs active post-crizotinib.	(23, 26)
BRAF V600E mutation	1-2%	Dabrafenib + Trametinib (Dual inhibition)	Vemurafenib + Cobimetinib, single-agent immunotherapy (if PD-L1 high)	Combination targeted therapy is standard; chemotherapy + immunotherapy is an alternative.	(28)
METex14 skipping	3-4%	Capmatinib or Tepotinib (Selective MET TKIs)	Savolitinib	High response rates. Safety profile includes edema and creatinine elevation.	(7, 22, 26)
RET fusion	1-2%	Selpercatinib or Pralsetinib (Selective RET TKIs)	/	Highly effective with durable responses. CNS active.	(28)
KRAS G12C mutation	~13%	Sotorasib or Adagrasib (Covalent inhibitors)	Platinum-doublet chemotherapy ± immunotherapy	Approved in second-line. First-line data maturing. STK11/KEAP1 co-mutations may predict poorer immunotherapy response.	(19, 29, 30)
NTRK fusion	<1%	Larotrectinib or Entrectinib (TRK inhibitors)	/	Tumor-agnostic approval. High response rates.	(28)
High PD-L1 (TPS ≥50%) no actionable driver	~30%	Pembrolizumab monotherapy (Anti-PD-1)	Cemiplimab, Atezolizumab (anti-PD-L1), or Pembrolizumab + Chemotherapy	PD-L1 is an imperfect biomarker. Combination chemo-immunotherapy may be preferred for high disease burden/rapid progression.	(8, 31)
Low/negative PD-L1 (TPS <50%) no actionable driver	~70%	Platinum-doublet Chemotherapy + Pembrolizumab (Non-squamous) or Nivolumab + Ipilimumab + 2 cycles of Chemotherapy (Squamous)	Platinum-doublet chemotherapy + Atezolizumab (non-squamous), or Chemotherapy alone	Standard for most without a driver. Benefit seen across PD-L1 subgroups, but greatest in PD-L1 ≥1%.	(9, 31)

## 2 Therapeutic innovations and resistance mechanisms in NSCLC

The therapeutic landscape of NSCLC is being reshaped by several innovative platforms that enhance precision and combat resistance. Antibody-drug conjugates (ADCs) exemplify this progress, with agents like HER3-DXd delivering cytotoxic payloads to EGFR-TKI resistant tumors and T-DXd combined with nivolumab showing promise in HER2-altered cancers (32, 33). These ADCs merge targeted delivery with potent cytotoxicity, offering new options where standard therapies fail. However, their distinct and sometimes notable toxicity profiles, such as the hematologic and gastrointestinal events seen with TROP-2-targeting ADCs or the interstitial lung disease risk with HER2-targeting agents, require careful management and influence their position in treatment sequences (3, 4, 32). Complementing ADCs, next-generation tyrosine kinase inhibitors (TKIs) continue to advance. Agents like lorlatinib for ALK-positive disease achieve unprecedented progression-free survival, including superior intracranial control, while selective inhibitors for MET exon 14 skipping (tepotinib) and KRAS G12C (glecirasib, divarasib) validate once “undruggable” targets (3, 19, 22). Yet, each TKI class carries a distinct toxicity spectrum, from lipid abnormalities with lorlatinib to edema with MET inhibitors, necessitating vigilant monitoring and affecting their suitability for combination strategies (5, 26, 34). Beyond traditional pharmacotherapy, novel modalities are emerging. Oligonucleotide therapies, such as FOXP3-targeting antisense oligonucleotides (ASOs), aim to modulate the immunosuppressive tumor microenvironment (TME), while nanoparticle systems seek to improve drug bioavailability (35, 36). Concurrently, T-cell engineering via therapies like afami-cel demonstrates the potential of cellular immunotherapy in specific solid tumors (17). The critical appraisal of these innovations must balance their promising efficacy against the practical challenges of their toxicity profiles, the optimal timing of their use within treatment algorithms, and their potential for synergistic combination.

### 2.1 Molecular profiling techniques and biomarker advances

**Figure 2:**
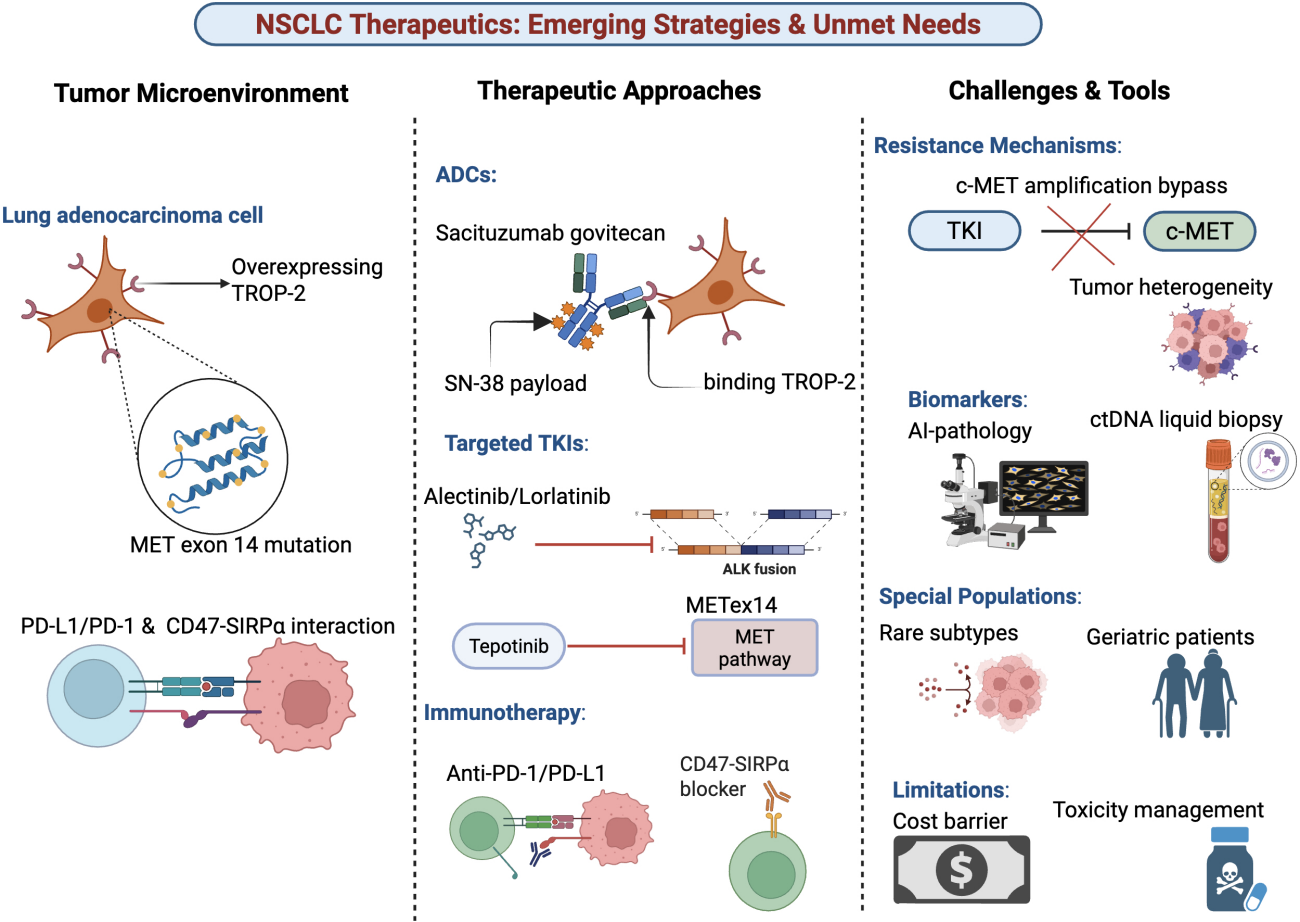
Emerging therapeutic strategies and unmet needs in non–small cell lung cancer. This schematic summarizes key features of the NSCLC therapeutic landscape across three domains. Left: Tumor microenvironment characteristics, highlighting lung adenocarcinoma cells with TROP-2 overexpression, MET exon 14 (METex14) alterations, and immune checkpoint interactions involving PD-1/PD-L1 and CD47–SIRPα signaling. Middle: Current and emerging therapeutic approaches, including antibody–drug conjugates (e.g., sacituzumab govitecan delivering the SN-38 payload via TROP-2 binding), targeted tyrosine kinase inhibitors for ALK fusions and METex14–driven signaling (e.g., alectinib, lorlatinib, tepotinib), and immunotherapeutic strategies targeting PD-1/PD-L1 and CD47–SIRPα pathways. Right: Ongoing challenges and enabling tools, encompassing resistance mechanisms such as c-MET amplification and tumor heterogeneity, biomarker development using AI-assisted pathology and circulating tumor DNA (ctDNA) liquid biopsy, considerations for special populations (rare molecular subtypes and geriatric patients), and practical limitations including cost barriers and toxicity management.

Accurate patient stratification is the cornerstone of precision oncology, driven by advances in molecular profiling. CGP using next-generation sequencing (NGS) detects actionable genomic alterations in a significant proportion of advanced solid tumors, expanding therapeutic opportunities beyond routine testing (37, 38). Liquid biopsy, particularly circulating tumor DNA (ctDNA) analysis, offers a non-invasive alternative for genotyping, resistance monitoring, and dynamic assessment of treatment response (39–41). The prognostic value of ctDNA dynamics, where clearance or early reduction correlates with improved outcomes, is a compelling advancement for real-time adaptation (10, 41). However, significant limitations impede universal implementation. The sensitivity of ctDNA assays is not absolute, and tissue-based profiling remains the gold standard for initial comprehensive assessment (39, 40). Furthermore, the clinical utility of CGP, while clear in increasing target detection, requires further validation regarding its definitive impact on survival outcomes and cost-effectiveness compared to standard testing algorithms (11, 12). Beyond genomics, transcriptomic and proteomic profiling are revealing predictive immune signatures and metabolic biomarkers, such as T-cell infiltration and circulating L-arginine levels, which may better predict immunotherapy response (42, 43). AI applied to histopathology shows nascent promise in predicting genetic mutations from routine tissue sections, potentially circumventing costly testing (9). Yet, its current performance is suboptimal for clinical adoption, lacking robust external validation across diverse populations and seamless integration into pathology workflows (11). These diagnostic tools, while powerful, face the dual challenges of rigorous biomarker validation and the creation of standardized, accessible clinical pathways (42). **Figure 2**.

### 2.2 Actionable genomic targets and overcoming therapeutic resistance

The expansion of actionable targets directly informs therapeutic strategy. For EGFR-mutant NSCLC, osimertinib is the established standard, with combinations like osimertinib plus ramucirumab showing further progression-free survival (PFS) benefit (18, 20). In ALK-positive cancer, lorlatinib sets a new benchmark for long-term disease control (3, 20). Targets such as KRAS G12C, METex14, and NTRK fusions now have effective inhibitors, enabling tumor-agnostic treatment approaches in some cases (28, 44). However, the durability of these responses is universally challenged by acquired resistance. Primary and secondary resistance arises through on-target mutations (EGFR C797S, ALK G1202R), off-target bypass pathway activation (MET, HER2, AXL amplification), and phenotypic transformation (NSCLC-to-SCLC transition) (26, 28). Immunotherapy resistance is driven by an immunosuppressive TME, with mutations in STK11/KEAP1 or metabolic alterations like low L-arginine contributing to immune evasion (29, 30, 43).

**Figure 3:**
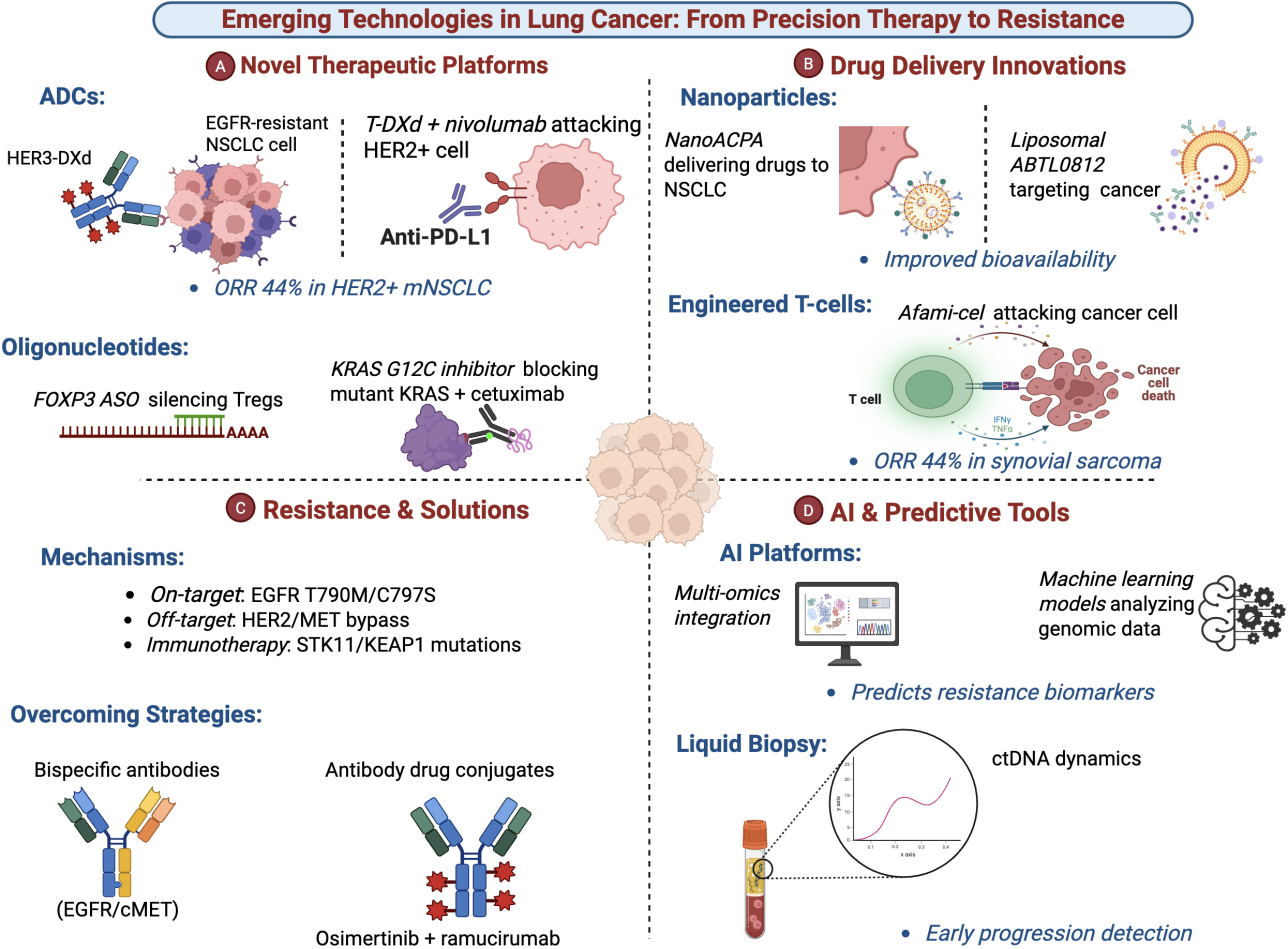
Emerging technologies in lung cancer: from precision therapy to resistance management. This figure highlights cutting-edge technological advances shaping modern lung cancer treatment, organized into four thematic panels. **(A)** Novel therapeutic platforms: Antibody–drug conjugates (e.g., HER3-DXd and T-DXd combined with nivolumab) targeting resistant and HER2-positive NSCLC, alongside oligonucleotide-based strategies such as FOXP3 antisense oligonucleotides to modulate regulatory T cells and KRAS^G12C^–directed inhibition combined with EGFR blockade. **(B)** Drug delivery innovations: Nanoparticle-based systems (e.g., NanoACPA) and liposomal formulations designed to improve tumor-specific drug delivery and bioavailability, as well as engineered T-cell therapies (e.g., afami-cel) mediating direct cancer cell killing. **(C)** Resistance and solutions: Key resistance mechanisms including on-target EGFR mutations (T790M/C797S), off-target bypass signaling via HER2/MET, and immunotherapy resistance linked to STK11/KEAP1 alterations, together with emerging strategies to overcome resistance using bispecific antibodies, antibody–drug conjugates, and rational combination therapies (e.g., osimertinib plus ramucirumab). **(D)** AI and predictive tools: Integration of multi-omics data and machine-learning platforms to predict resistance biomarkers, complemented by liquid biopsy approaches using circulating tumor DNA dynamics for early detection of disease progression and therapeutic response monitoring.

Overcoming this resistance requires innovative strategies. Next-generation TKIs are designed to target specific resistance mutations, as seen with ficonalkib for ALK G1202R (26). ADCs offer a potent strategy against tumors with bypass resistance by delivering payloads independently of the original signaling pathway (32, 33). Proactive combination therapies, such as osimertinib with ramucirumab or divarasib with cetuximab, aim to delay resistance by simultaneously blocking primary targets and escape routes (18, 44). Emerging approaches include bispecific antibodies (targeting EGFR/c-MET), synthetic lethality strategies (PARP inhibitors in homologous recombination-deficient tumors), and autologous T-cell therapies designed to overcome antigen loss (17, 45, 46). A critical component of managing resistance is longitudinal monitoring via ctDNA, which can detect molecular relapse ahead of clinical progression and guide adaptive intervention (28, 40, 41). The future of NSCLC therapy lies in deepening the molecular characterization of resistance and deploying these sequential and combinatorial strategies within a framework of dynamic biomarker monitoring (47). **Table 3**; **Figure 3**.

**Table 3:** Biological mechanisms, resistance, biomarkers, or novel therapeutic strategies.

Study objective	Methodology	Main findings	Conclusion	Reference
Investigate resistance mechanisms of HER3-DXd	Phase 1 trial (102 patients)	Acquired ERBB3/TOP1 mutations at progression; Median OS 16.2 months	ERBB3/TOP1 mutations drive resistance	(33)
Evaluate T-DXd + nivolumab in HER2+ metastatic breast cancer and urothelial carcinoma	Phase 1b/2 trial	ORR: HER2+ breast cancer 65.6%, urothelial carcinoma 36.7%; ILD incidence 20.7% (breast)	Activity in HER2+ tumors; ILD requires monitoring	(32)
Test AZD8701 (FOXP3 ASO) ± durvalumab	Phase 1 trial (63 patients)	FOXP3 knockdown achieved; Stable disease ≥16 weeks: 24.4% (monotherapy)	Feasible with FOXP3 suppression	(36)
Assess divarasib + cetuximab in KRAS G12C+ colorectal cancer	Phase 1b trial (29 patients)	ORR 62.5% (TKI-naïve); ERBB3/TOP1 resistance mutations	ERBB3/TOP1 mutations confer resistance	(44)
Evaluate NanoACPA in NSCLC	Preclinical xenografts + pharmacokinetics in mice	NanoACPA bioavailability 5.5× > free ACPA; Tumor regression via Akt/PI3K/JNK pathway	Improved delivery and efficacy; A promising clinical candidate	(35)
Identify biomarkers for pembrolizumab in PD-L1<50% NSCLC	Multiomics analysis (flow cytometry RNA-seq/metagenomics) in 65 patients	High NK cells/CD56dimCD16+ (HR 0.56) and CD14dimCD16+ monocytes (HR 0.52) linked to longer PFS	Immune subsets predict pembrolizumab benefit	(31)
Analyze ctDNA dynamics in ensartinib-treated ALK+ NSCLC	Phase 2 trial + ctDNA analysis (180 patients)	TP53 mutations/high ctDNA VAF correlated with worse OS; ctDNA clearance predicted better outcomes	ctDNA and TP53 status are prognostic	(41)
Assess plasma L-arginine as a biomarker for immune checkpoint inhibitors	Retrospective analysis + preclinical model	Low L-arginine linked to worse OS (HR 3.03); Preclinical models showed high L-arginine improved tumor rejection (85.7% vs. 23.8%)	L-arginine predicts immune checkpoint inhibitors efficacy; correlates with immunosuppression	(42)
Evaluate next-generation sequencing-guided therapy in cancers of unknown primary	Phase 2 trial (97 patients)	1-year survival 53.1%; Targetable EGFR in 5.2% (durable PFS >6 months)	Next-generation sequencing identifies actionable targets in cancers of unknown primary	(28)
Study impact of STK11/LKB1 mutations in KRAS+ NSCLC	Phase 2 IFCT TASTE trial (134 patients)	STK11/LKB1 mutations associated with shorter DFS (HR 3.85) vs. KRAS+ alone	STK11/LKB1 mutations worsen prognosis	(30)
Define molecular determinants of PD-L1 blockade	Retrospective analysis (366 patients; PD-L1/TMB/RNA-seq)	NSCLC/UC share cell cycle/DDR signatures; CDKN2A associated with response	Molecular heterogeneity requires indication-specific approaches	(48)
Assess impact of AGAs on neoadjuvant immune checkpoint inhibitors in resectable NSCLC	Subanalysis (44 patients)	AGAs linked to higher treatment failure (HR 5.51) and reduced pathological regression	AGAs predict immune checkpoint inhibitors resistance	(49)
Test biomarker-directed therapies + durvalumab	Phase 2 umbrella trial (268 patients)	ATM-altered: ORR 26.1%; Median PFS 5.8 months vs. 2.7 months (others)	Durvalumab-ceralasertib effective in ATM-altered NSCLC	(50)
Evaluate toripalimab + docetaxel in EGFR TKI-resistant NSCLC	Phase 2 trial + ctDNA dynamics (33 patients)	ctDNA clearance at 6 weeks predicted ↑PFS (HR 100, P = 0.005)	ctDNA dynamics predict efficacy	(51)

## 3 Diagnostic, biomarker, and implementation advances in NSCLC

The translation of therapeutic innovation into clinical benefit is governed by diagnostic pathways, biomarker validation, and the realities of healthcare delivery. This section examines the integration of molecular testing into clinical algorithms, the challenges of implementation, and strategies to optimize management across diverse care contexts.

### 3.1 Molecular testing workflows and stage-directed treatment algorithms

Precision oncology in NSCLC is initiated through structured molecular testing workflows. For newly diagnosed advanced NSCLC, current guidelines mandate comprehensive biomarker testing to guide first-line therapy. This includes assessment of EGFR, ALK, ROS1, BRAF, NTRK, METex14, RET, and KRAS G12C mutations, alongside PD-L1 expression. The integration of NGS, either via tissue-based CGP or liquid biopsy, is increasingly advocated to efficiently identify these and other rare actionable targets (11, 37, 38). As illustrated in a proposed molecular testing workflow (see **Figure 2**), the choice between tissue and plasma-based testing is influenced by tissue availability, tumor burden, and urgency for results, with reflex to the alternative modality following an inconclusive initial result (39, 40, 52).

This biomarker data directly informs stage-specific treatment algorithms. For early-stage resectable disease, the standard of care involves surgery with or without adjuvant therapy, though neoadjuvant and perioperative immunotherapy combinations are now emerging as new standards, with their efficacy potentially modulated by specific actionable genomic alterations (53, 54). The management of unresectable stage III disease has been revolutionized by consolidative durvalumab following chemoradiation, establishing a benchmark against which novel combinations are being tested (50). For stage IV disease, treatment selection is stratified by driver mutation status and PD-L1 expression, as summarized in a biomarker-directed first-line therapy (see **Table 2**). Notably, the positioning of emerging agents is continuously evolving within these sequences. For instance, ADCs such as HER3-DXd are specifically under investigation for patients with EGFR-mutant NSCLC who have developed acquired resistance to EGFR TKIs (33). Similarly, T-DXd combined with immunotherapy is being evaluated in patients with HER2-mutant or HER2-overexpressing advanced NSCLC (32), and TROP-2-targeting ADCs like datopotamab deruxtecan are being developed for advanced, refractory non-oncogene-addicted NSCLC (3, 4). Next-generation TKIs, such as lorlatinib for ALK-positive disease, are firmly established in the first-line setting for their respective molecular subsets (3, 20). Understanding this algorithmic context and the precise indications for novel therapies is essential for evaluating where they may fill unmet needs or displace current standards.

### 3.2 Clinical implementation challenges and access disparities

Despite established guidelines, the implementation of precision medicine faces profound systemic challenges. Access to comprehensive molecular testing remains inconsistent; in some real-world cohorts, only about half of advanced NSCLC patients receive complete biomarker profiling (52). Restricted gene panels miss a significant proportion of actionable alterations compared to broader CGP, potentially denying patients effective therapies (55, 56). While liquid biopsy can mitigate tissue scarcity issues, its sensitivity is not perfect, and it may fail to detect alterations present in tissue (39, 40).

Therapeutic access is further hindered by reimbursement barriers and infrastructural limitations. Even when actionable alterations are identified, a staggering proportion of patients; estimated at 66-89%, do not receive a genomically matched therapy due to drug access issues, restrictive clinical trial eligibility, or clinical deterioration (38, 56, 57). Molecular tumor boards (MTBs) have been established to interpret complex genomic data and recommend therapies, succeeding in doing so for 19-61% of cases (39, 58). However, MTBs face operational hurdles, including lengthy turnaround times for CGP reports, variant interpretation challenges, and integration into busy clinical workflows. Furthermore, the added cost of CGP (€1.5K-€3.9K per patient) and a lack of NGS infrastructure in resource-limited settings create significant geographic and socioeconomic disparities in care (56, 57, 59). These barriers collectively ensure that the promise of precision oncology remains unrealized for a majority of eligible patients, underscoring an urgent need for standardized, cost-effective, and equitable implementation models (55). **Table 4**.

**Table 4:** Emerging therapies discussed in this review and their potential clinical position.

Therapeutic class/strategy	Specific agents/targets	Key mechanism of action	Current development status/evidence	Potential clinical positioning	Reference
Antibody-Drug Conjugates (ADCs)	TROP-2 targeting (e.g., Sacituzumab govitecan, Datopotamab deruxtecan)	Cytotoxic payload delivery to TROP-2 overexpressing tumor cells.	Phase III trials in refractory NSCLC; promising activity post-chemotherapy/immunotherapy.	Later-line therapy for advanced, refractory non-oncogene-addicted NSCLC.	(3, 4)
	HER3-DXd (Patritumab deruxtecan)	Topoisomerase I inhibitor delivery to HER3-expressing cells.	Phase I/II in EGFR-TKI resistant EGFR-mutant NSCLC.	Later-line therapy for EGFR-mutant NSCLC with acquired resistance to available TKIs.	(33)
	T-DXd (Trastuzumab deruxtecan) + Nivolumab	Anti-HER2 ADC combined with PD-1 blockade.	Phase Ib/II in HER2-expressing metastatic cancers.	Potential strategy for HER2-overexpressing or mutated NSCLC, likely in later lines or selected frontline combinations.	(32)
Next-generation targeted therapies	Glecirasib (KRAS G12C inhibitor)	Irreversible covalent inhibition of KRAS G12C.	Phase IIb in KRAS G12C+ NSCLC.	Second-line+ therapy for KRAS G12C+ NSCLC; first-line evaluation ongoing.	(19)
	Divarasib (KRAS G12C inhibitor) + Cetuximab	KRAS inhibition combined with EGFR blockade to prevent feedback signaling.	Phase Ib in KRAS G12C+ colorectal cancer; rationale applicable to NSCLC.	Potential strategy to overcome or prevent adaptive resistance to KRAS G12C monotherapy.	(44)
	Ficonalkib (ALK TKI)	Next-gen ALK inhibitor designed to target resistant mutations (e.g., G1202R).	Phase I/II in ALK+ NSCLC post prior TKIs.	Later-line therapy for ALK+ NSCLC with resistance to earlier-generation ALK TKIs.	(26)
Novel immunotherapy strategies (beyond PD-1/PD-L1)	CD47-SIRPα Axis Inhibitors	Blockade of “don’t eat me” signal to enhance macrophage-mediated phagocytosis.	Early-phase trials in solid tumors, including NSCLC.	Potential combination partner with PD-1/PD-L1 inhibitors, especially in immunologically cold tumors.	(8)
	FOXP3-targeting ASO (AZD8701)	Antisense oligonucleotide reducing immunosuppressive Treg activity.	Phase I alone/with durvalumab in advanced solid tumors.	Immunomodulatory agent for combination strategies to overcome TME-driven immunotherapy resistance.	(36)
Other modalities	Bispecific Antibodies (e.g., EGFR/cMET)	Simultaneous engagement of two antigens (e.g., EGFR and cMET) to block bypass resistance.	Preclinical/early clinical development.	Strategy to overcome bypass resistance in EGFR-mutant or other driven cancers.	(45)
	Autologous T-cell Therapy (e.g., Afami-cel)	Engineered T-cells targeting MAGE-A4 antigen.	Phase I in MAGE-A4+ solid tumors (e.g., synovial sarcoma).	Potential for shared antigen-positive NSCLC, likely after exhaustion of standard therapies.	(17)
	Nanoparticle Delivery (e.g., NanoACPA)	Nanocarrier to improve drug bioavailability and targeting.	Preclinical proof-of-concept in NSCLC xenografts.	Platform technology to enhance delivery and efficacy of various therapeutic payloads.	(35)
AI-driven strategies	AI for Mutation Prediction	Prediction of genomic alterations (e.g., EGFR, BRAF) from H&E-stained pathology slides.	Retrospective validation studies; performance suboptimal for clinical use.	Potential future triage tool to prioritize cases for molecular testing, pending rigorous validation.	(9, 11)

### 3.3 Optimizing care for special populations and future directions

Optimizing NSCLC management requires tailored approaches for special populations often underrepresented in clinical trials. Elderly patients, who constitute a large proportion of the NSCLC population, require careful assessment beyond chronological age. Comprehensive geriatric assessments evaluating functional status, comorbidities, polypharmacy, and social support are crucial for determining fitness for therapy. Evidence suggests that while TKIs like osimertinib are effective and tolerable in older adults with EGFR mutations, combination regimens may incur excessive toxicity, favoring monotherapy approaches (13). Similarly, for patients with active central nervous system metastases, selecting agents with high central nervous system penetration (lorlatinib, osimertinib) is a critical component of treatment planning (3, 31, 49).

Ethnic and regional genetic differences also influence disease management. The higher prevalence of EGFR mutations in Asian populations (40-55%) compared to Caucasians (10-15%) justifies different empirical treatment approaches and trial designs (50, 51). Furthermore, research on rare histological subtypes (hepatoid adenocarcinoma) is inherently limited, often relying on retrospective data to inform management, which typically involves surgery for localized disease and platinum-based chemotherapy with or without immunotherapy for advanced stages (14).

**Figure 4:**
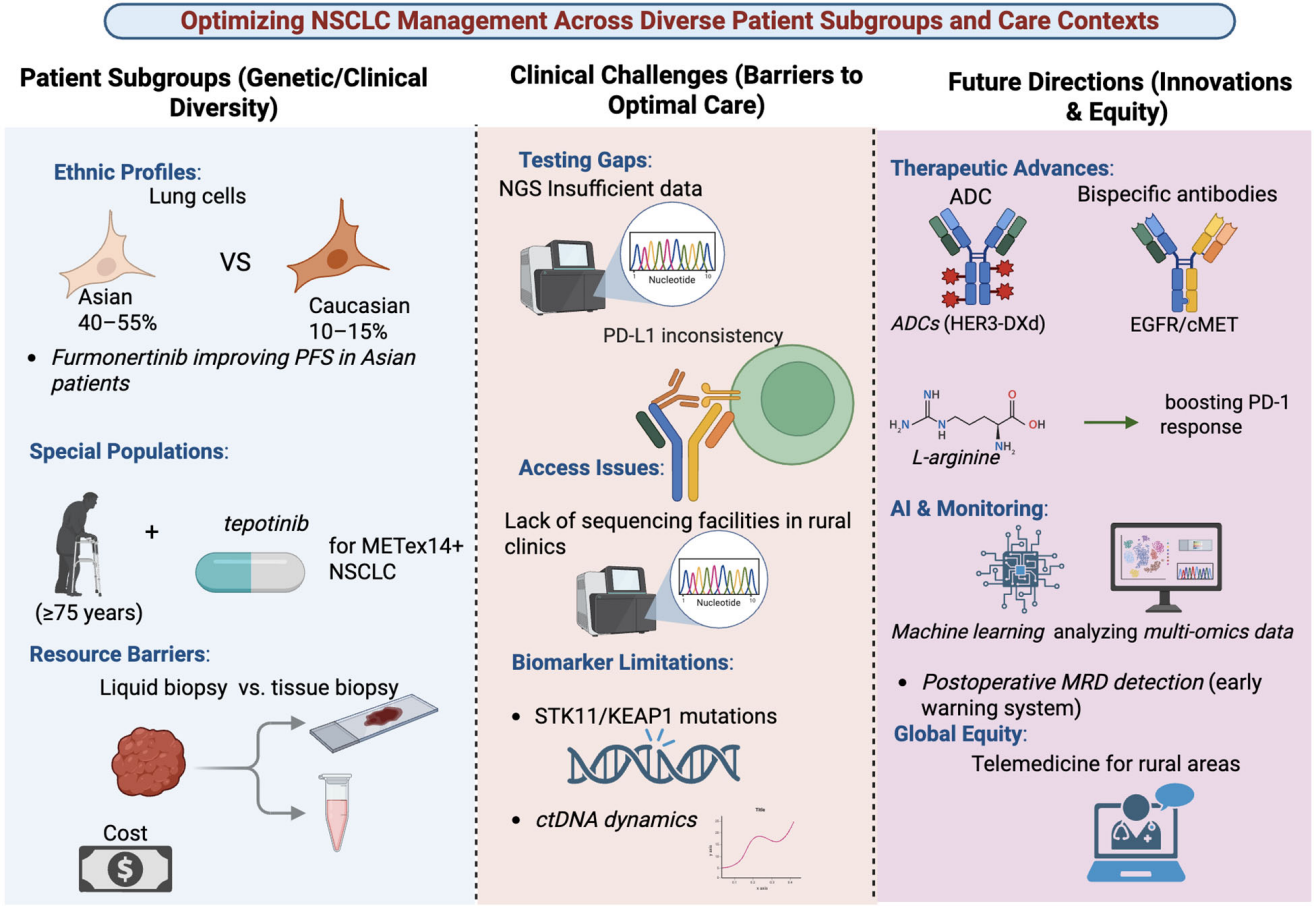
Optimizing NSCLC management across diverse patient subgroups and care contexts. This schematic illustrates key determinants of equitable and effective non–small cell lung cancer (NSCLC) care. Left: Genetic, clinical, and ethnic heterogeneity influencing treatment response, including differences in mutation prevalence across populations, special considerations for elderly patients and METex14-positive disease, and resource-related barriers affecting access to tissue versus liquid biopsy. Middle: Major clinical challenges to optimal care, encompassing testing gaps in next-generation sequencing (NGS), variability in PD-L1 assessment, limited access to molecular diagnostics, and biomarker constraints such as STK11/KEAP1 alterations and dynamic ctDNA changes. Right: Future directions aimed at innovation and equity, highlighting advances in antibody-based therapeutics and bispecific antibodies, AI-driven multi-omics analysis and minimal residual disease (MRD) monitoring, and telemedicine strategies to expand access to precision oncology in resource-limited and rural settings.

Future directions must address these multifaceted challenges through convergent strategies. Diagnostically, efforts should focus on validating and standardizing dynamic biomarkers like ctDNA for minimal residual disease detection and early progression (27, 52). Therapeutically, the development of novel platforms, such as bispecific antibodies, next-generation ADCs, and cellular therapies, must be coupled with pragmatic combination and sequencing studies (17, 21, 39). Operationally, deploying cost-effective NGS panels, expanding MTB access, and developing equitable reimbursement models are essential to democratize precision medicine (24, 26). Finally, dedicated research initiatives incorporating geriatric assessments and international registries for rare subtypes are needed to generate robust evidence for all patient subgroups. By integrating these advances in diagnostics, therapeutics, and health systems, the field can progress toward more personalized, effective, and equitable NSCLC care for every patient (41, 53). **Table 5**, **Figure 4**.

**Table 5:** Strengths and limitations of novel approaches in NSCLC.

Novel approach	Key strengths	Challenges	Reference
Antibody-Drug Conjugates (ADCs)	• Targeted Potency: Deliver highly cytotoxic payloads directly to tumor cells via antigen-specific targeting, improving therapeutic index. • Activity in Refractory Disease: Demonstrate efficacy in tumors resistant to chemotherapy, TKIs, and immunotherapy. • Bypass Resistance: Can target tumors with bypass resistance mechanisms independent of original oncogenic signaling.	• Toxicity: Exhibit unique and sometimes severe adverse events (e.g., neutropenia, interstitial lung disease) requiring expert management. • Antigen Heterogeneity/Loss: Efficacy depends on stable antigen expression; target heterogeneity or downregulation can lead to resistance. • Resistance Mechanisms: Emerging on-target (e.g., ERBB3/TOP1 mutations for HER3-DXd) and off-target resistance are being characterized.	(3, 4, 32, 33)
Next-generation Tyrosine Kinase Inhibitors (TKIs)	• Superior Efficacy & CNS Control: Offer significantly improved progression-free survival and intracranial activity vs. earlier-generation agents. • Targeting “Undruggable” Drivers: Validate inhibition of previously difficult targets (e.g., KRAS G12C, METex14). • High Selectivity: Reduced off-target effects compared to earlier multi-kinase inhibitors.	• Acquired Resistance: Inevitable development of on-target (e.g., ALK G1202R) and bypass resistance. • Class-Specific Toxicities: Carry distinct adverse event profiles (e.g., lipid abnormalities, edema) impacting tolerability and combination potential. • Access & Cost: High cost and reimbursement barriers limit global accessibility.	(22, 26)
Novel immunotherapy strategies (beyond PD-1/PD-L1)	• Diverse Mechanisms: Target alternative immune checkpoints (e.g., CD47-SIRPα) or immunosuppressive cell populations (e.g., Tregs) to overcome primary resistance. • Combination Potential: Offer rational partners for PD-1/PD-L1 inhibitors to convert “cold” tumors. • Metabolic Modulation: Targeting metabolites like L-arginine addresses a key axis of TME immunosuppression.	• Biomarker Gaps: Lack of validated predictive biomarkers for patient selection beyond PD-L1. • Safety Profile: Novel immunomodulatory mechanisms carry unique toxicities (e.g., anemia, thrombocytopenia). • Clinical Validation: Most approaches are in early-phase trials; comparative efficacy vs. standards is unknown.	(8, 36, 43)
Comprehensive genomic profiling (CGP) & liquid biopsy	• Broad Target Discovery: Identifies a wider range of actionable genomic alterations compared to single-gene tests, including rare fusions. • Non-Invasive Monitoring: ctDNA enables real-time assessment of treatment response, early detection of resistance, and minimal residual disease monitoring. • Overcomes Tissue Limitations: Provides an option when tissue is inadequate or unobtainable.	• Access & Cost: Expensive with significant reimbursement hurdles; limited availability in resource-poor settings. • Interpretation & Actionability: Complex reports and lack of targeted therapies for some alterations limit clinical utility. • Sensitivity & Specificity: ctDNA assays have imperfect sensitivity, especially in low-volume disease, and may not capture tumor heterogeneity fully.	(52, 56, 57)
Artificial intelligence (AI) in pathology	• Predictive Potential: Can predict genetic mutations from routine H&E slides, potentially reducing cost and turnaround time. • Objective Quantification: Digitally quantifies features like tumor-infiltrating lymphocytes for reproducible biomarker assessment. • Integration with Multi-Omics: AI platforms can integrate histologic, genomic, and clinical data for novel biomarker discovery.	• Limited Clinical Validation: Performance is suboptimal for standalone clinical use; requires large-scale, multi-center validation. • Generalizability: Models trained on specific populations/institutions may not generalize broadly. • Workflow Integration: Significant technical and logistical barriers to implementation in routine pathology practice.	(9, 11, 42)
Bispecific antibodies & cellular therapies	• Overcomes Antigen Loss/Heterogeneity: Engages multiple antigens or uses cellular mechanisms to circumvent common resistance pathways. • Precision Targeting: Engineered T-cell therapies offer high specificity for tumor-associated antigens. • Immune Engagement: Bispecific antibodies can directly recruit and activate immune cells at the tumor site.	• Complex Manufacturing & Logistics: Autologous cellular therapies are patient-specific, costly, and have complex supply chains. • Safety: Risk of severe toxicity (e.g., cytokine release syndrome, neurotoxicity). • Limited Antigen Targets: Applicability is restricted to patients expressing the specific target antigen (e.g., MAGE-A4), often a small subset.	(17, 45)

## 4 Conclusion

NSCLC research stands at a pivotal inflection point, marked by unprecedented therapeutic innovation yet challenged by persistent implementation gaps. The treatment landscape has expanded dramatically beyond chemotherapy and initial targeted agents, driven by breakthroughs in ADCs such as TROP-2-targeting sacituzumab govitecan and datopotamab deruxtecan, as well as HER3-DXd, which deliver potent cytotoxic payloads to refractory tumors, including EGFR-TKI-resistant and non-oncogene-addicted disease. Simultaneously, highly selective next-generation TKIs such as lorlatinib, tepotinib/savolitinib, and glecirasib/divarasib offer superior efficacy and central nervous system control, redefining standards of care for oncogene-driven subsets. Immunotherapy continues to evolve beyond PD-1/PD-L1, exploring novel targets such as CD47-SIRPα blockade and metabolic modulation such as L-arginine supplementation to overcome immunosuppressive TME.

However, this remarkable progress is counterbalanced by significant, multifaceted limitations. Acquired resistance to targeted therapies, driven by on-target mutations such as EGFR C797S and ALK G1202R, bypass pathway activation, or lineage plasticity, remains a fundamental biological hurdle. While strategies such as proactive combination therapies and next-generation ADCs show promise in circumventing resistance, their long-term durability and optimal sequencing require further validation. Biomarker development lags behind therapeutic innovation. PD-L1 expression is an imperfect predictor, and validated biomarkers for newer agents and immunotherapy resistance are urgently needed. The integration of liquid biopsy for ctDNA dynamics offers a powerful non-invasive tool for real-time monitoring, early progression detection, and minimal residual disease assessment, but its prognostic utility demands prospective validation in diverse clinical pathways.

Crucially, translating research advances into equitable patient benefit faces substantial systemic barriers. Robust data for special populations, particularly older adults and those with rare subtypes such as hepatoid adenocarcinoma of the lung, remain scarce, necessitating dedicated trials incorporating geriatric assessments. While CGP detects a broader range of actionable targets, its real-world clinical utility and cost-effectiveness are hampered by inconsistent access, reimbursement hurdles, lengthy turnaround times, and complex interpretation. Only 11-34% of eligible patients ultimately receive matched targeted therapies, with disparities exacerbated in resource-limited settings. Molecular tumor boards improve target identification but face operational challenges. Furthermore, the toxicity profiles of newer agents and novel combinations necessitate careful management and longer-term safety data.

Looking ahead, the future of NSCLC management hinges on several converging strategies. First, overcoming resistance requires deepening the molecular characterization of relapse mechanisms and developing effective sequential or combinatorial regimens leveraging ADCs, bispecific antibodies, and cellular therapies. Second, biomarker refinement is essential, utilizing AI-driven multi-omics integration to discover predictive signatures and validating ctDNA dynamics for real-time adaptation. Third, pragmatic implementation must address accessibility by streamlining CGP workflows, developing cost-effective panels, establishing equitable reimbursement models, and expanding liquid biopsy use globally. Fourth, dedicated research must focus on special populations through geriatric-focused trials and international registries for rare subtypes. Finally, tumor-agnostic approaches and interception strategies hold promise for expanding treatable populations.

In essence, while the therapeutic arsenal for NSCLC has never been more potent or precise, realizing its full potential demands a concerted effort to dismantle biological, technological, and systemic barriers. Success will depend on integrating deep molecular science with pragmatic solutions for biomarker validation, accessible profiling, tailored toxicity management, and inclusive trial design. By bridging these gaps, the field can transform current momentum into sustained, equitable improvements in survival and quality of life for all NSCLC patients. The path forward is complex, but the foundation for transformative progress is firmly established.
